# Disease-specific Nutritional Physical Therapy: A Position Paper by the Japanese Association of Rehabilitation Nutrition (Secondary Publication)

**DOI:** 10.31662/jmaj.2021-0202

**Published:** 2022-03-25

**Authors:** Tatsuro Inoue, Izumi Takeuchi, Yuki Iida, Kohei Takahashi, Fumihiko Nagano, Shinjiro Miyazaki, Kengo Shirado, Yoshihiro Yoshimura, Ryo Momosaki, Keisuke Maeda, Hidetaka Wakabayashi

**Affiliations:** 1 Department of Physical Therapy, Niigata University of Health and Welfare, Niigata, Japan; 2 Department of Rehabilitation, Suizenji Tohya Hospital, Kumamoto, Japan; 3 Department of Physical Therapy, Toyohashi SOZO University School of Health Sciences, Aichi, Japan; 4 Department of Rehabilitation, Tamura Surgical Hospital, Kanagawa, Japan; 5 Department of Rehabilitation, Kumamoto Rehabilitation Hospital, Kumamoto, Japan; 6 Rehabilitation Center, KKR Takamatsu Hospital, Kagawa, Japan; 7 Department of Rehabilitation, Iizuka Hospital, Fukuoka, Japan; 8 Center for Sarcopenia and Malnutrition Research, Kumamoto Rehabilitation Hospital, Kumamoto, Japan; 9 Department of Rehabilitation Medicine, Mie University Graduate School of Medicine, Mie, Japan; 10 Department of Geriatric Medicine, Hospital, National Center for Geriatrics and Gerontology, Aichi, Japan; 11 Department of Rehabilitation Medicine, Tokyo Women’s Medical University Graduate School of Medicine, Tokyo, Japan

**Keywords:** Exercise, Nutritional disorders, Nutritional therapy, Resistance training

## Abstract

Nutritional disorders diminish the effectiveness of physical therapy. The pathogenesis of nutritional disorders, such as sarcopenia, frailty, and cachexia, differs from disease to disease. Disease-specific nutrition can maximize the function, activity, participation, and quality of life for patients undergoing physical therapy, a practice known as nutritional physical therapy. Understanding and practicing disease-specific nutritional physical therapy is essential to meet patients’ diverse needs and goals with any disease. Thus, the physical therapist division of the Japanese Association of Rehabilitation Nutrition, with advice from the Japanese Society of Nutrition and Swallowing Physical Therapy, developed this review. It discusses the impact of disease-specific nutritional physical therapy on sarcopenia and frailty in community-dwelling older adults, obesity and metabolic syndrome, critical illness, musculoskeletal diseases, stroke, respiratory diseases, cardiovascular diseases, diabetes, renal disease, cancer, and sports.

## 1. Introduction

Malnutrition, sarcopenia, frailty, and cachexia impair the health status and reduce the effectiveness of physical therapy. It is necessary to deal with nutritional disorders according to their pathogenesis as they differ from disease to disease. Skeletal muscle atrophy occurs early in the disease in patients with critical illness due to increased muscle breakdown caused by severe invasion. In stroke patients, dysphagia and motor paralysis lead to malnutrition and sarcopenia. Osteoarthritis and osteoporotic fractures indicate different nutritional disorders in patients with musculoskeletal diseases. Chronic heart failure, chronic obstructive pulmonary disease, and cancer cause cachexia by pathogenic mechanisms characteristic of each disease and lead to a poor prognosis. Therefore, it is necessary to understand disease-specific nutritional physical therapy to deal with these nutritional disorders. We aim to provide a basis for practicing disease-specific nutritional physiotherapy through this review.

## 2. Disease-specific Nutritional Physical Therapy (Table 1)

### Sarcopenia and frailty in the community-dwelling older adults

Sarcopenia and frailty are geriatric syndromes that should not be ignored during physical therapy. Sarcopenia is a skeletal muscle disease that causes age-related loss of muscle mass, muscle weakness, and decreased physical function, leading to falls, hospitalization, and death ^(1)^. Sarcopenia is categorized as primary and secondary sarcopenia. Primary sarcopenia indicates sarcopenia with no apparent cause other than aging. Secondary sarcopenia indicates the presence of causes other than aging. It is caused by disease, inactivity, inadequate intake of energy or protein ^(2)^. The prevalence of sarcopenia in community-dwelling older adults is approximately 10%-20% in Japan ^(3), (4)^, and it is a risk factor for disability and death ^(4)^. The Asian Working Group for Sarcopenia criteria ^(5)^ and its 2019 version ^(6)^ have been widely used in Japan. Frailty is a comprehensive concept that indicates the physical, psychological, and social aspects of aging ^(7), (8)^. The Fried criteria ^(7)^ evaluate physical frailty, and the prevalence of physical frailty is approximately 10% in Japan ^(9), (10), (11)^. The diagnostic criteria of social frailty are not standardized, but it is a risk factor for disability ^(12)^, depression ^(13)^, and death ^(12)^. Thus, comprehensive interventions based on understanding the multiple aspects of frailty are needed.

**Table 1.** Disease-specific Nutritional Physical Therapy.

Disease	Disease-specific nutritional physical therapy
Sarcopenia and frailty in the community-dwelling older adults	Physical therapy for older people with sarcopenia and frailty includes a combination of resistance training, aerobic and balance exercises, and nutrition. Amino acid (EAA), and leucine metabolites such as β-hydroxy-β-methylbutyrate (HMB) and creatine, is effective for muscle protein synthesis^(16), (17)^.
Obesity and metabolic syndrome	Nutritional therapy for obesity and metabolic syndrome aims to increase muscle mass and decrease body fat mass simultaneously ^(24)^. To reduce 1 kg of stored fat, 7,500 kcal must be consumed. To increase muscle mass, protein should be unrestricted, and resistance training and protein intake should be combined. Aerobic exercise is effective for reduction of fat mass ^(25), (26)^.
Critically ill	Prevention of ICU-AW is an important intervention. Within 3-5 days after ICU admission, avoid over-feeding and gradually increase protein intake to 1.3 g/kg/day and calories to 70% of predicted levels ^(129)^. Start early mobilization with adequate protein intake and moderate energy expenditure by exercise, as muscle protein degradation increases due to hypercatabolism. After 5 days, nutrition should be maintained at a protein intake of at least 1.2 g/kg/day to induce muscle protein anabolism in collaboration with exercise stimulation. The exercise load should be 40% of the maximum load.
Musculoskeletal diseases	Emphasizing protein intake throughout the entire surgical process (pre- and postsurgical periods) reduces muscle atrophy and loss of function due to increased muscle protein catabolism and immobilization after orthopedic surgery ^(130)^. Protein intake of 1.2-2.0 g/kg/day is considered for the rehabilitation period following major surgery. For obese osteoarthritis patients, a combination of energy restriction (estimated energy expenditure −300-1000 kcal), meal replacement supplements with protein, resistance training, and aerobic exercise improves function and relieves pain^(131)^.
Stroke	Nutritional interventions include adjusting food texture and initiating oral intake early in patients with mild dysphagia. Tube feedings early and percutaneous endoscopic gastrostomy are recommended for patients who require enteral feedings for more than 28 days ^(132)^ in patients with severe dysphagia. Rehabilitation includes early mobilization, swallowing training, and assessment of eating posture. Administration of fortified nutritional supplements, a combination of leucine-enriched amino acid intake, and rehabilitation effectively improve sarcopenia and ADL ^(57), (58)^.
Respiratory diseases	Nutritional therapy such as dietary advice and fat and/or protein-enriched supplementation for stable COPD patients increased body weight, muscle mass, 6-min walk distance, and health-related QOL ^(71)^. Small and frequent oral intake is effective in avoiding dyspnea. The combination of exercise therapy such as resistance training and walking exercises and nutrition therapy effectively increases body weight and muscle mass and improves exercise tolerance, especially in patients with malnutrition^(71)^.
Cardiovascular diseases	In the therapeutic strategies for cardiac cachexia, comprehensive cardiac rehabilitation is useful, including appropriate heart failure medications, nutrition therapy, and exercise. Aerobic exercise training counteracts skeletal muscle wasting in addition to improving exercise tolerance. Resistance training is also recommended for cardiovascular disease patients with frailty and sarcopenia. In patients with chronic heart failure, protein intake of 1.2-1.5 g/kg and caloric supplementation based on 25-30 kcal/kg depending on the degree of stress ^(133)^ should be combined with exercise therapy.
Diabetes	Aerobic exercise and resistance training or a combined approach reduce the risk of developing type 2 diabetes and improve cardiovascular disease risk factors ^(134)^. Nutritional therapy optimizes total energy intake (25-35 kcal/target body weight/day) and corrects nutrient imbalances such as limiting saturated fatty acids.
Kidney disease	Aerobic exercise and resistance training are recommended to improve exercise tolerance and QOL. In nondialysis patients with severe renal dysfunction, exercise intensity is adjusted according to age and physical function ^(135)^. Energy and protein intake according to the severity of kidney disease and sarcopenia. In patients with low L-carnitine levels, the administration of L-carnitine maintains and improves exercise tolerance and muscle mass ^(136)^.
Liver disease	Nutritional therapy (energy: 35-40 kcal/kg/day, protein: 1.3-1.5 g/kg/day) such as BCAA supplementation and late evening snacks are recommended ^(137)^. Aerobic exercise, resistance training, or a combined approach is effective, but it needs to be careful with decreasing hepatic blood flow during and after exercise.
Cancer	A multidisciplinary approach, including physical and nutritional therapy, is recommended to improve the response to treatment, prognosis, and QOL ^(44)^. The combination of aerobic exercise and resistance training effectively improves fatigue and QOL. Nutritional physical therapy needs to be considered based on cancer treatment or palliative care.
Sports	Athletes’ physical activity decreases immediately after injury or surgery, while rehabilitation and training for returning to competition often involve high-intensity exercise ^(106)^. Therefore, exercise energy expenditure and energy stores need to be considered in terms of energy needs ^(106)^. Because female athletes are prone to low energy availability (LEA), physical therapists should conduct nutritional screening including LEA (weight, eating disorder, bone density/damage, amenorrhea, etc.) ^(110), (138)^, when conducting rehabilitation.
Anorexia	The American Psychiatric Association guidelines recommend starting at 30-40 kcal/kg/day and increasing 70-100 kcal/kg/day during the weight gain phase ^(112)^. The National Institute for Clinical Excellence guidelines for anorexia nervosa set a weekly weight gain goal of 0.5-1 kg for inpatients and 0.5 kg for outpatients, adding approximately 3,500-7,000 kcal/week ^(113)^. Controlled physical activity ^(116)^ and low-intensity resistance training ^(117)^ are safe and beneficial for restoring body composition, maintaining bone density, and decreasing anxiety ^(115)^.
Depression	The Mediterranean diet is associated with a lower risk of depression ^(120)^. Eicosapentaenoic acid and docosahexaenoic acid are effective in treating mood disorders, impulse control disorders, and psychotic disorders ^(121)^. Both aerobic and resistance exercise are beneficial for depression, and the higher the intensity and volume of exercise, the more effective ^(123), (124)^.

Resistance training (RT) combined with nutritional therapy with amino acids is effective for sarcopenia in older adults ^(14), (15), (16), (17)^. Physical therapy, such as RT, aerobic exercise, and balance exercise ^(18)^, combined with nutritional therapy, mainly amino acids, effectively prevents functional impairment.

### Obesity and metabolic syndrome

Obesity and metabolic syndrome lead to a decline in physical function and quality of life (QOL). In Japan, obesity is defined as a body mass index (BMI) of ≥25 kg/m^2^; this is lower than the World Health Organization definition of ≥30 kg/m^2^ ^(19)^. The prevalence of obesity in Japan is approximately 30% of men aged 40-60 years and more than 20% of women aged ≥50 years ^(20)^. Obesity is also associated with impaired mobility, instrumental activities of daily living (IADL), and QOL ^(21)^.

Sarcopenic obesity has a stronger impact on negative health outcomes than obesity alone in older adults; it has a higher risk for IADL decline, frailty, falls, hip fractures, gait disturbance, cardiovascular disease, and death than obesity alone ^(22), (23)^. Insulin resistance due to intramuscular fat accumulation, proinflammatory cytokines such as interleukin (IL)-6, and vitamin D deficiency accelerate the loss of muscle mass and decrease muscle strength, leading to physical dysfunction ^(24), (25)^.

Nutritional physical therapy for obesity and metabolic syndrome aims to increase muscle mass and decrease body fat simultaneously. In a systematic review, combined interventions of RT and protein intake improved lean body mass and lower extremity muscle strength compared with RT alone in obese older adults ^(26)^. Nutritional physical therapy for obesity includes combined RT and nutritional interventions, as well as aerobic exercises ^(27), (28)^.

### Critically ill patients

In critically ill patients, such as those with sepsis, a state of systemic hypercatabolism exists, causing various biological reactions, including the production of inflammatory cytokines and increased immune activity. Skeletal muscle proteins break down into amino acids used for endogenous energy supply in the form of protein synthesis and glucose regeneration. As a result of the degradation of skeletal muscle, which accounts for most of the protein stored in the body, muscle mass and bodyweight decrease. Myofibrillar proteins (actin and myosin), accounting for 60%-70% of the muscle proteins, are degraded ^(29)^. In critically ill patients, the amount of muscle proteins lost per day is 250 g, equivalent to 750-1,000 g of muscle mass ^(30)^. This amount can be translated into approximately four times the daily loss of skeletal muscle mass due to short periods of fasting ^(31)^. Skeletal muscle dysfunction in critically ill patients admitted to the intensive care unit (ICU) is called ICU-acquired weakness (ICU-AW). It worsens the patient’s prognosis and health-related QOL even if they are eligible for discharge ^(29)^. ICU-AW complicates about half of all critically ill patients with sepsis, multiple organ failure, or prolonged mechanical ventilation management ^(32)^. In addition, the development and progression of ICU-AW is caused by the combined and synergistic effect of invasive medication, hypoactivity, and malnutrition ^(33)^. Therefore, it is extremely important to reduce these factors as countermeasures for ICU-AW.

Other factors contributing to skeletal muscle dysfunction in critically ill patients include inactivity and malnutrition. Inactivity causes a decrease in muscle protein synthesis and an increase in protein degradation, leading to skeletal muscle atrophy. The decrease in muscle protein synthesis is observed early in the inactive period (6-24 h) and is prolonged ^(34)^. Bed rest for 5 days decreases the size of muscle fibers by 3.5%-10% and muscle strength by 9%-13% ^(35)^. In patients with severe disease, inadequate energy intake leads to a worse prognosis. Insufficient energy causes increased catabolism, which leads to further weight loss as protein and fat are broken down and consumed. In a study of the relationship between cumulative energy balance and prognosis, survival was poor in patients with multiple organ failure at levels of less than 10,000 kcal ^(36)^.

An appropriate combination of nutrition and exercise therapy should be used to reduce catabolic effects and promote recovery. A recovery program with a focus on physical therapy and nutrition is recommended in the early stages of ICU admission ^(37)^. In the early stages of the disease, the patient should receive enteral nutrition with high protein and leucine-containing amino acids, early mobilization and exercise therapy, and neuromuscular electrical stimulation therapy ^(38)^. It is important to correct the imbalance between catabolic and anabolic effects in the acute phase through rehabilitation nutrition intervention to promote the recovery of physical functions.

### Musculoskeletal diseases

Patients with musculoskeletal diseases often suffer from malnutrition and sarcopenia. In hip fracture patients, the prevalence of malnutrition and sarcopenia was 7%-26% and 11%-76.4%, respectively ^(39)^. Malnutrition and sarcopenia diminish functional recovery and increase mortality and morbidity ^(39), (40)^. Sarcopenic obesity is due to age-associated loss of muscle mass and increased fat mass, obesity-associated inflammation, and pain-associated inactivity ^(41)^. The development and progression of sarcopenic obesity lead to the development and progression of osteoarthritis ^(41)^. Sarcopenic obesity delays functional recovery in patients after hip arthroplasty ^(42)^.

Nutritional physical therapy is effective in patients with musculoskeletal diseases and coexisting nutritional disorders. Nutritional therapy for older patients with hip fracture undergoing rehabilitation reduces mortality and improves muscle strength and ADL ^(43), (44)^. The updated clinical practice guidelines for rehabilitation nutrition recommended enhanced nutritional therapy in patients with hip fractures undergoing rehabilitation ^(44)^. It is especially useful to combine physical therapy and nutrition therapy, such as oral nutritional supplements and individual nutritional counseling. In obese patients with osteoarthritis, a combination of diet and exercise therapy is useful for weight loss, functional improvement, and pain relief ^(45), (46)^. Furthermore, energy restriction (estimated energy expenditure of −300-1000 kcal) combined with meal replacement supplements, RT, and aerobic exercise is useful ^(46), (47)^.

### Stroke

Stroke patients suffer from malnourishment, mainly due to impaired consciousness and dysphagia. The prevalence of malnutrition in patients with stroke ranges from 6.1% to 62% ^(48)^, and is associated with an increased incidence of infections, pressure ulcers, gastrointestinal bleeding, longer hospital stays, and mortality during hospitalization. In recovery-phase stroke patients, malnutrition diminishes ADL improvement ^(49)^.

Stroke-related sarcopenia is caused by denervation, muscle atrophy, and dysphagia ^(50)^. The prevalence of sarcopenia in patients with stroke is 53.6% ^(51) (52)^ and sarcopenia leads to reduced ADL recovery, dysphagia, and a lower rate of home discharge ^(53)^ compared with nonsarcopenia. Decreased muscle cross-sectional area ^(54), (55), (56)^ and increased subcutaneous and intramuscular fat mass ^(56)^ caused by paralysis is characteristic of strokes.

In the case of nutritional problems in patients with stroke, the combined intervention of exercise and nutrition therapy effectively improves clinical outcomes. The updated clinical practice guidelines for rehabilitation nutrition recommend enhanced nutrition therapy for patients with stroke undergoing rehabilitation ^(44)^. The combination of leucine-enriched amino acid intake and rehabilitation effectively increases skeletal muscle mass and improves ADL in stroke patients with sarcopenia in the convalescent rehabilitation wards^(57), (58)^.

### Respiratory Diseases

Chronic respiratory disease, mainly chronic obstructive pulmonary disease (COPD), is associated with a higher incidence of weight loss and sarcopenia, which leads to exacerbations and death. The prevalence of malnutrition in COPD patients is 24.6% and 45.0%, according to the European Society for Clinical Nutrition and Metabolism ^(59)^ and the Global Leadership Initiative on Malnutrition criteria ^(60)^, respectively, which is associated with increased hospitalization and mortality. Unintentional progressive weight loss is the strongest independent risk for mortality ^(61)^. The prevalence of sarcopenia in patients with COPD was 15.5% ^(62)^-21.6% ^(63)^, which is associated with a decreased predicted forced expiratory volume in the first second, exercise tolerance, and QOL compared with nonsarcopenic patients ^(62)^. In addition, reduced muscle strength ^(64)^ and fat-free mass ^(65)^ are independent predictors of mortality in patients with COPD. Low skeletal muscle mass is a risk factor for mortality in patients with idiopathic pulmonary fibrosis ^(66), (67)^.

Sarcopenia in respiratory diseases is caused by aging, decreased activity, chronic inflammation, and malnutrition; malnutrition and sarcopenia overlap in COPD patients ^(68)^. Patients with COPD have chronically elevated inflammatory cytokines ^(69)^. Muscle strength and skeletal muscle mass were significantly correlated with high-sensitivity tumor necrosis factor (TNF), IL-6, and aging, and lower BMI, cardiovascular complications, and elevated high-sensitivity TNF were associated with sarcopenia in COPD patients ^(70)^.

The combination of exercise and nutrition therapy effectively increases body weight and muscle mass and improves exercise tolerance, especially in patients with malnutrition. Nutritional supplementation for stable COPD patients increases weight gain, muscle mass, 6-min walk distance, and health-related QOL ^(71)^. The combination of nutritional supplementation and exercise resulted in greater weight gain, with greater improvement in the malnutrition group than in the nutritional supplementation alone group. In the statement of nutritional assessment and therapy in COPD from the European Respiratory Society, nutritional intervention is probably effective in malnourished patients, most likely when combined with exercise ^(72)^. Recently, the definitions and diagnostic criteria for respiratory sarcopenia and sarcopenic respiratory disability have been developed ^(73)^. Future studies should examine the prevalence and impact of interventions, including nutritional physical therapy, in older patients with respiratory diseases.

### Cardiovascular diseases

The obesity paradox occurs in patients with cardiovascular disease. Weight loss, cachexia, and loss of muscle mass are risk factors for poor prognosis. Increasing BMI improves cardiovascular and all-cause mortality in patients with coronary artery disease ^(74)^ and heart failure ^(75)^. Unintentional progressive weight loss is an independent predictor of all-cause mortality in patients with chronic heart failure ^(76)^. The prevalence of cachexia and sarcopenia in ambulatory patients with heart failure was 18.8% and 21.3%, respectively ^(77)^; in addition, 6.7% of patients had comorbid cachexia and sarcopenia ^(77)^. Skeletal muscle loss in patients with heart failure exacerbates other clinical symptoms, leading to decreased QOL, prolonged hospitalization, increased frequency of readmission, and reduced survival ^(78)^. Sarcopenia is an independent predictor of 1-year mortality in patients with heart failure with reduced ejection fraction and preserved ejection fraction ^(79)^.

To improve cachexia in heart failure, a combination of appropriate medications, nutritional therapy, and exercise is useful. Nutritional therapy increases body weight; it reduces all-cause mortality and hospital readmission in patients with heart failure coexisting with malnutrition or cardiac cachexia, but the strength of the evidence is poor ^(80)^. Systemic inflammation is associated with increased skeletal muscle breakdown, leading to decreased skeletal muscle mass and strength ^(81)^. Exercise reduces inflammatory cytokines and improves exercise tolerance in patients with heart failure ^(82)^. Aerobic exercise training counteracts skeletal muscle wasting in the therapeutic strategies of cardiac cachexia ^(83)^. Comprehensive cardiac rehabilitation, including nutrition, exercise, and medication, has been shown to increase nutritional intake and improve muscle strength and walking speed, even in patients with sarcopenia hospitalized with cardiovascular disease ^(84)^.

### Diabetes, kidney disease, and liver disease

Many patients with diabetes, kidney disease, and liver disease have nutritional disorders that lead to increased complications and mortality ^(85), (86), (87)^. The mechanisms of the onset of sarcopenia due to hyperglycemia have not been elucidated, but an accumulation of advanced glycation end-product ^(88)^, changes in the skeletal muscle extracellular matrix, and reduced insulin action have been suggested as possible factors ^(89)^. Decreased physical function and loss of muscle mass are attributed to inflammation and atherosclerosis syndrome ^(90)^, cardio-renal anemia syndrome, and abnormal bone mineral metabolism associated with chronic kidney disease ^(91)^. Liver disease is often associated with sarcopenia due to elevated ammonia and low branched-chain amino acid levels caused by protein-energy malnutrition ^(92), (93)^.

The combination of nutritional and physical therapy is effective for the treatment of diabetes and liver disease. A Cochrane review of patients at high risk of developing type 2 diabetes reported that a combination of nutritional therapy and physical activity intervention reduced or delayed the onset of type 2 diabetes compared with each intervention alone ^(94)^. A systematic review of patients with cirrhosis showed that exercise and nutritional therapy effectively improve sarcopenia ^(95)^.

### Cancer

Most patients with cancer who require rehabilitation are malnourished or sarcopenic ^(96)^. Weight loss is associated with shorter survival, lower QOL ^(97)^, poor adherence to anticancer therapy, and increased side effects in patients with cancer ^(98), (99)^. Furthermore, patients with gastrointestinal cancers, such as esophageal cancer, frequently develop malnutrition and skeletal muscle weakness, which leads to increased postoperative complications and mortality ^(100), (101), (102)^.

The effects of comprehensive interventions, including exercise and nutrition therapy, have been reported in cancer patients. The updated clinical practice guidelines for rehabilitation nutrition recommend enhanced nutritional care for patients undergoing (or after) anticancer treatment and rehabilitation (certainty of evidence: moderate; recommendation level: weak) ^(44)^. In patients with upper gastrointestinal cancer, preoperative physical rehabilitation, and nutritional interventions, mainly whey protein, improved preoperative and postoperative exercise tolerance than usual care ^(103)^. Physical rehabilitation and nutritional intervention in the perioperative period reportedly improved postoperative muscle strength in patients with sarcopenic older gastric cancer ^(104)^. Combined exercise and nutritional interventions have shown safety and high compliance in older pancreatic cancer and nonsmall cell lung cancer patients undergoing chemotherapy ^(105)^. Thus, nutritional physical therapy is recommended in the perioperative period, including preoperatively and during chemotherapy, in patients with cancer.

### Sports

It is important for physical therapists to assess athletes’ nutritional status and cooperate with dietitians as needed. The combined application of nutritional care management is beneficial in physical therapy for injured athletes ^(106), (107)^． Immobilization of the affected area, decreased activity, and increased muscle protein catabolism due to trauma or postsurgical inflammation cause loss of skeletal muscle mass and weakness, affecting the possibility of returning to play ^(106), (107)^. In addition, limited or decreased physical activity may increase body fat deposition. The combination of rehabilitation and nutritional management reduces postoperative complications, minimizes disability and loss of skeletal muscle mass, and maximizes the possibility of returning to play ^(106)^. In nutritional management, the following are considered: metabolic fluctuation during trauma or postoperatively and energy consumption in rehabilitation ^(106)^. Physical therapy for returning to play often involves high-intensity exercise, which increases energy expenditure. A negative energy balance decreases skeletal muscle mass. Therefore, physical therapists need to cooperate with dietitians and consider optimizing energy requirements, nutrient timing, and use of selected nutritional supplements.

In other cases, athletes may experience low energy availability (LEA) because of increased energy expenditure due to exercise or extreme dietary restrictions. The prevalence of LEA in athletes is 22%-58%; it is present in both sexes but is particularly common in women ^(108)^. Para-athletes with spinal cord injury also have a high risk of LEA ^(109)^. Long-term LEA can cause health problems such as emaciation, osteoporosis, amenorrhea, and anemia and affect sports performance ^(108), (109)^. Previous studies have shown that physical therapists involved in athletic rehabilitation lack knowledge and awareness of LEA and nutritional status ^(110)^. Adjusting the physical therapy program and the load considering the nutritional status leads to the prevention of LEA and its complications (e.g., stress injury and stress fracture) ^(110)^. Physical therapists need to use nutritional screening to assess the nutritional status of athletes and cooperate with dietitians and clinicians to prevent and improve LEA^(110)^.

### Anorexia nervosa

Anorexia nervosa (AN) is a complex, severe disease characterized by abnormal eating behavior. Severe malnutrition with excessive weight loss causes cardiovascular, gastrointestinal disorders, and metabolic abnormalities ^(111)^. Fat intake is often reduced in patients with AN compared with healthy controls, while the carbohydrate and protein intake results are not consistent ^(111)^. The American Psychiatric Association guidelines for AN recommend starting at 30-40 kcal/kg/day (approximately 1,000-1,600 kcal/day) and increasing gradually to 70-100 kcal/kg/day during the weight gain phase ^(112)^. National Institute for Clinical Excellence guidelines for AN recommend 0.5-1 kg as a weekly weight gain goal for inpatients and 0.5 kg for outpatients. Thus, approximately 3,500-7,000 kcal per week are recommended to achieve these goals ^(113)^. It is necessary to select a variety of foods without avoiding essential nutrients. During the initiation of nutritional therapy, attention should be paid to refeeding syndrome caused by rapid refeeding. Refeeding syndrome is caused by hypophosphatemia, hypokalemia, and hypomagnesemia, which lead to arrhythmia, congestive heart failure, and hypotension ^(111)^.

It is controversial how to manage physical activity and exercise in patients with AN. Many patients with AN have hyperactivity ^(114)^. Hyperactivity leads to increased energy requirements to achieve weight gain, worse clinical outcomes, longer hospital stays, and relapse of AN ^(115)^. Thus, rest has been a priority for patients with AN. However, controlled physical activity ^(116)^ and low-intensity resistance training ^(117)^ can be performed safely and may be beneficial in restoring lean mass, maintaining bone mineral density, and decreasing anxiety ^(115)^. It is unclear what level of physical activity and exercise is safe and beneficial in patients with AN.

### Depression

Depression is a psychiatric disorder closely related to nutritional factors in the prevention and treatment phases. Meta-analysis shows that obesity and metabolic syndrome lead to the onset of depression, and conversely, depression is accompanied by overeating and decreased physical activity, which causes obesity and metabolic syndrome ^(118), (119)^. Mediterranean diet is associated with a lower risk of depression ^(120)^. The American Psychiatric Association recommends supplementation of eicosapentaenoic acid and docosahexaenoic acid among n-3 (omega-3) unsaturated fatty acids for mood disorders, impulse control disorders, and psychotic disorders ^(121)^.

Increasing physical activity and exercise are effective for depression. A Cochrane systematic review reported that the effect of exercise on depression was mild to moderate ^(122)^. Both aerobic and anaerobic exercise is effective in reducing depression symptoms ^(123), (124)^. For major or minor depression in subjects aged 60 years or older, high-intensity (80% maximum load) progressive RT improved depression symptoms compared with low-intensity (20% maximum load) ^(125)^. In addition, for mild to moderate major depression, the 17.5 kcal/kg/week exercise group reduced depression symptoms, while the 7 kcal/kg/week group was as effective as the control group ^(126)^. Both aerobic and resistance exercise are beneficial for depression, and the higher the intensity and volume, the more effective ^(127)^.

## 3. Future Perspectives

This review discusses the results of studies that provide evidence for the beneficial effects of disease-specific nutritional physiotherapy; however, the evidence for nutritional physical therapy is not abundant. Further clinical research is needed to provide evidence for disease-specific nutritional physiotherapy.

This is the secondary English version of the original Japanese manuscript for “Disease-specific nutritional physical therapy: A position paper by the Japanese Association of Rehabilitation Nutrition” ^(128)^.

## Article Information

### Conflicts of Interest

None

### Acknowledgement

We would like to thank the Japanese Society of Nutrition and Swallowing Physical Therapy for their advice in developing this position paper. We are also grateful for public comments on this paper.

### Author Contributions

Substantial contributions to the conception or design of the work: Tatsuro Inoue, Izumi Takeuchi, Yuki Iida, Kohei Takahashi, Fumihiko Nagano, Shinjiro Miyazaki, Kengo Shirado, Yoshihiro Yoshimura, Ryo Momosaki, Keisuke Maeda, and Hidetaka Wakabayashi

Drafting the work: Tatsuro Inoue, Izumi Takeuchi, Yuki Iida, Kohei Takahashi, Fumihiko Nagano, Shinjiro Miyazaki, Kengo Shirado, Yoshihiro Yoshimura, Ryo Momosaki, Keisuke Maeda, and Hidetaka Wakabayashi

Final approval of the version to be published: Tatsuro Inoue, Izumi Takeuchi, Yuki Iida, Kohei Takahashi, Fumihiko Nagano, Shinjiro Miyazaki, Kengo Shirado, Yoshihiro Yoshimura, Ryo Momosaki, Keisuke Maeda, and Hidetaka Wakabayashi

Agreement to be accountable for all aspects of the work in ensuring that questions related to the accuracy or integrity of any part of the work are appropriately investigated and resolved: Tatsuro Inoue, Izumi Takeuchi, Yuki Iida, Kohei Takahashi, Fumihiko Nagano, Shinjiro Miyazaki, Kengo Shirado, Yoshihiro Yoshimura, Ryo Momosaki, Keisuke Maeda, and Hidetaka Wakabayashi

### Approval by Institutional Review Board (IRB)

Not applicable.

* The editors-in-chief of the Journal of the Japanese Association of Rehabilitation Nutrition and JMA journal have given permission for secondary publication of this position paper.

* This article is based on a study first reported in the Journal of the Japanese Association of Rehabilitation Nutrition. 2021; 5(2):p 217-225 (Japanese) ^(128)^.

Journal articles published ahead of issue (print): Inoue T et al. Disease-specific nutritional physical therapy: A position paper by the physical therapist section of the Japanese Association of Rehabilitation Nutrition. The Journal of the Japanese Association of Rehabilitation Nutrition. 2021.

The original version is available at https://sites.google.com/site/jsrhnt/ポジションペーパー?authuser=0.
